# Tonsillar Plasmacytoma: clues on magnetic resonance imaging

**DOI:** 10.1186/s12880-018-0261-9

**Published:** 2018-06-18

**Authors:** İrfan Çelebi, Gülpembe Bozkurt, Nedim Polat

**Affiliations:** 1 Department of Radiology, Sisli Hamidiye Etfal Education and Research Hospital, Istanbul, Turkey; 2 Department of Otolaryngology, Acıbadem University Faculty of Medicine, Istanbul, Turkey; 3 Department of Pathology, Beykent University Medical Faculty, Istanbul, Turkey; 4 Department of Otolaryngology-Head and Neck Surgery, Acıbadem Üniversitesi Atakent Hastanesi, Halkalı Merkez Mahallesi, Halkalı Altınşehir İstanbul Cd. No:16, 34303 Küçükçekmece, İstanbul, Türkiye

**Keywords:** Plasmacytoma, Multiple myeloma, Magnetic resonance imaging, Asymmetric tonsillar enlargement

## Abstract

**Background:**

Malignant plasma cell proliferation may present as a disseminated disease (multiple myeloma), a solitary plasmacytoma of bone, or an extramedullary plasmacytoma of soft tissue. The latter plasmacytomas represent approximately 3% of all plasma cell proliferations, and 80% develop in the head-and-neck region. The unexpected clinical presentation of such masses may be present.

**Case presentation:**

Here, we report a rare case of primary tonsillar plasmacytoma in a 42-year-old female. The patient presented with asymmetric tonsillar hypertrophy that was resistant to antibiotherapy. Upon further workup, we found no evidence of multiple myeloma or light-chain disease. The patient underwent surgery and, at the last follow-up, exhibited no evidence of such disease.

**Conclusions:**

In adults presenting with asymptomatic tonsillar enlargement, the possibility of submucosal masses should be considered, thus encouraging the radiologist to evaluate crypts within the palatine tonsil on a postcontrast MRI, besides enlargement and signal change.

## Background

Extramedullary plasmacytomas (EMPs) are rare, comprising about 3% of all plasma cell neoplasms. Such plasmacytomas exhibit bone marrow plasma cell infiltration, < 5% nucleated cells, and no evidence of myeloma [1]. EMPs usually develop in the head-and-neck region (80%), with the nasopharynx and sinonasal cavities being the most common sites [2, 3]. However, EMPs may originate in any region where lymphoid deposits are found, including submucosal tissues of the pharynx, larynx, and oral cavity, and nonmucosal regions such as the thyroid gland [1, 4]. One published series found that < 5% of EMPs arose in the pharyngeal tonsils and oropharynx [5, 6].

Patients typically present in the fifth to seventh decades of life with localized submucosal masses or swellings, and symptoms reflecting compression and obstruction of local structures. In this paper, we present a rare case of a solitary tonsillar EMP presenting as a slightly asymmetric persistent enlargement of the tonsils. As far as we know, there are no reports in radiologic literature that illustrate magnetic resonance imaging (MRI) contribute to the diagnosis of tonsillary plasmacytoma.

## Case presentation

**Fig. 1:**
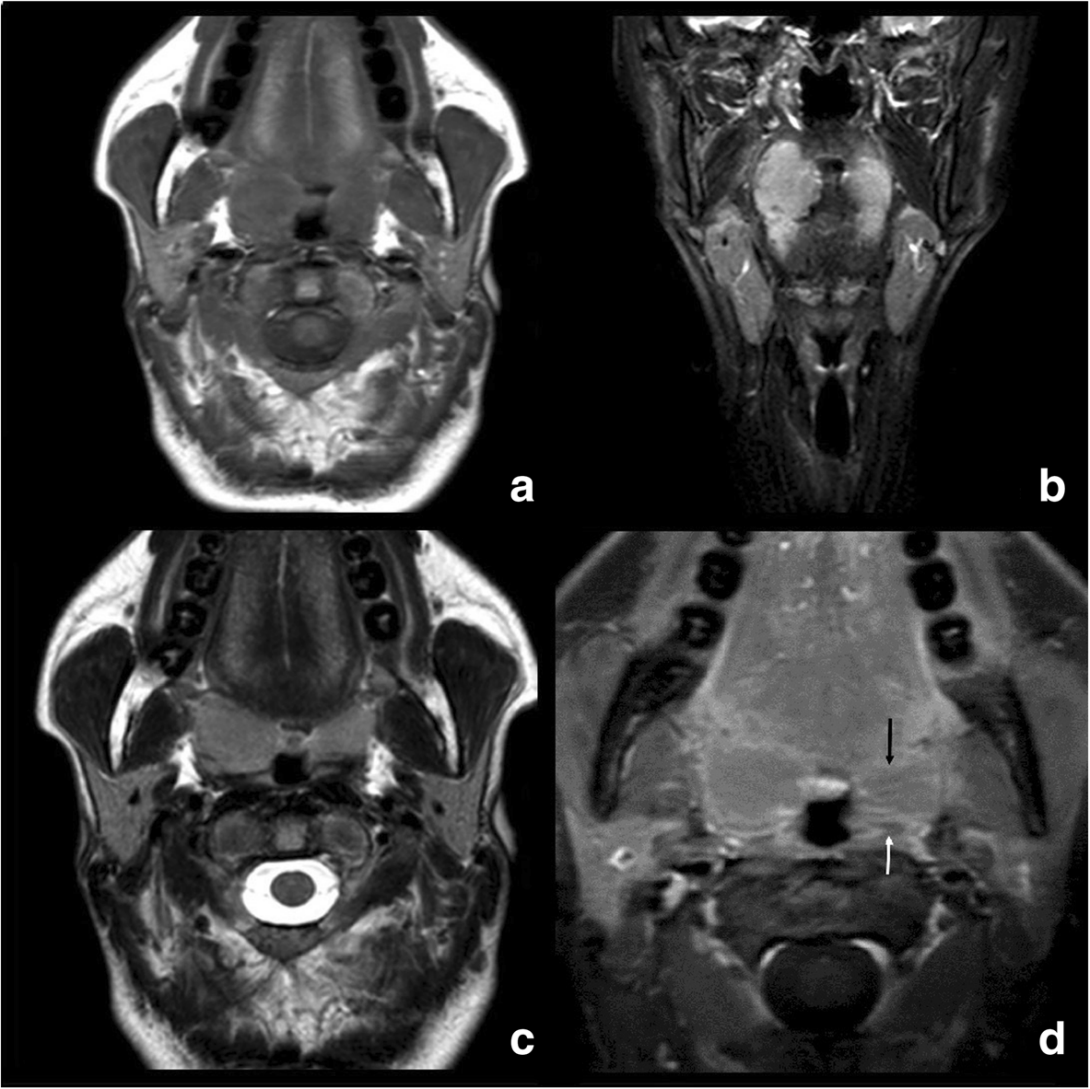
Axial noncontrast T1 (**a**), coronal STIR (**b**), Axial T2-weighted (**c**) and postgadolinium T1-weighted with fat saturation (**d**) MR images. Note the obliteration of the right palatine tonsillar crypts combined with linear horizontal enhancement to the left (arrow in **d**)

A 42-year-old female was seen by a primary care physician, whose examination of the oral cavity revealed smooth bilaterally enlarged tonsils, with the right tonsil being slightly larger than the left. No surface abnormality was evident. She was prescribed two courses of amoxicillin but did not improve. She was then referred to our otorhinolaryngology department. Her medical history was unremarkable. We found no unexplained cervical lymphadenopathy, no significant systemic symptom, no malignancy, and no immunocompromise. All laboratory parameters were within normal limits. Magnetic resonance (MR) images of the palatine tonsils did not reveal any obvious mass lesion. Axial noncontrast T1 (600/8/2 [TR/TE/NEX]) and coronal STIR MR (5700/80/1) images of the palatine tonsils showed that the right tonsil was larger than the left. No mass or abnormal T2 prolongation (suggestive of a tumor) was evident. An axial T2-weighted image (4400/100/2) and a postgadolinium T1-weighted image with fat saturation (550/8/1) also failed to reveal any mass in the right tonsil, but on postcontrast MRI, the left tonsil showed mucosal crypts with linear enhancement, while on the right side the crypts were partly obliterated by a large mass and did not display enhancement (Fig. 1).

**Fig. 2:**
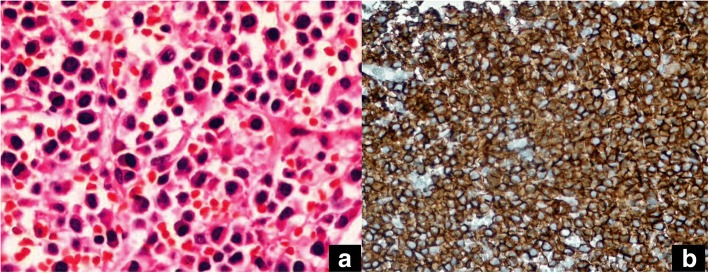
Plasma cells with eccentric nucleus and abundant cytoplasm (hematoxylin eosin × 40) (**a**), Diffuse cytoplasmic, focal membranous CD138 staining on the plasma cells (streptavidinebiotin peroxidase × 400) (**b**)

The patient underwent complete surgical resection of the right tonsil because of a possible malignancy. Histological examination of the specimen revealed diffuse sheets of monomorphous plasmacytoid cells with abundant, eosinophilic finely granular cytoplasm, and eccentric nuclei. Occasional binucleated and pleomorphic cells with giant nuclei and prominent nucleoli were also observed, as were mitotic figures. No amyloid deposition was apparent. Immunohistochemically, the plasma cells were diffusely immunoreactive for the λ light chain (DAKO, Glostrup, Denmark), IgG (DAKO), and CD43 (DAKO); partially positive for epithelial membrane antigen (DAKO); but negative for the κ light chain (DAKO), IgA (DAKO), IgM (DAKO), and CD20 (DAKO). Additional immunohistochemical staining showed that the cells were positive for the plasma cell markers CD138, MUM-1, and CD56. Thus, we diagnosed a plasmacytoma (Fig. 2).

A metastatic workup was performed to search for multiple myeloma/light chain disease. Laboratory testing revealed the absence of anemia, and the serum calcium, albumin, total protein, and creatinine levels were normal. The urine lacked Bence-Jones protein. The bone survey was negative. Bone marrow biopsy revealed < 5% plasma cells, ruling out plasmacytoma of the bone. No monoclonal gammopathy was evident, precluding an immunofixation disorder. The kappa-to-lambda ratio was normal. No adjuvant treatment was indicated, and the patient remains clinically free of disease after 6 months of follow-up.

## Discussion

The 2016 World Health Organization classification of lymphoid neoplasms divided plasma cell tumors into plasma cell myeloma (multiple myeloma [MM]), solitary plasmacytoma of bone [SBP], and extraosseous plasmacytoma [7]. EMP is rare in adults and constitutes a form of non-Hodgkin’s lymphoma [8]. EMP involving the tonsil is unusual and has only been sporadically reported in the literature [6, 9].

It is common for tonsillar asymmetry to raise suspicion of malignancy, often indicating the need for a tonsillectomy. This is especially true in adults aged 18 and above where MRI evaluations are frequently required. Whether tonsillar asymmetry is the only clinical finding that truly reflects a risk of EMP has been frequently discussed in the otorhinolaryngologic literature. Some authors have suggested that all unilaterally enlarged tonsils should be excised to counter possible malignancies even when no other suspicious feature is noted at presentation [10, 11]. In 1999, Reiter et al. concluded that asymmetric (but otherwise normal) tonsils were suggestive of a significant risk of lymphoma, which tends to grow submucosally and therefore occasionally presents with asymmetry only; histological examination was also recommended [2]. Conversely, other researchers recommend watchful waiting when asymmetry is the only abnormal clinical finding because of the low incidence of malignancy in unilaterally enlarged tonsils [12, 13]. Several authors have reported that unilateral tonsillar enlargement may be attributable to mucosal asymmetry in the tonsillar pillars or differences in the depths of the tonsillar fossae. When considering management options in such patients, the risk associated with unnecessary tonsillectomy should be balanced against that of delayed diagnosis of a tonsillar malignancy. As far as we know, there are no radiologic contributions reported in radiologic literature to diagnose submucosal tonsillar masses with the exception of tonsillary enlargement and signal change.

MM is a known sequela of EMP. The rate of conversion of EMP to MM is lower than that of other plasma cell neoplasms such as SPB; the reported rates range from 11 to 33% over 10 years [14]. The risk of conversion is highest in the first 2 years, but conversion has been recorded up to 15 years after diagnosis. Therefore, a lack of clinical suspicion in our patient was associated with a risk of delayed diagnosis and the possible unavailability of optimal therapy.

Today, imaging modalities such as computed tomography and MR imaging are used to stage and assess the responses of malignant lymphomas, rather than for lymphoma diagnosis per se [15]. No report has yet described how to image plasmacytomas optimally. The only striking finding in our patient was obliteration of the right palatine tonsillar crypts which did not show enhancement, as compared to linear enhancement in the crypts seen in the normal left tonsil. This case report emphasizes that disappearing of tonsillar crypts on postcontrast MRI is the most effective diagnostic sign for extramucosal intratonsillar masses which have no signal differentiation on non-contrast MR examination. However, this feature is not exclusive of EMP ---but helped decide that such a tonsil needed further investigation due to strong possibility of neoplasia. And that further corroboration of this feature is needed in more cases.

The differential diagnoses should include malignant lymphoma (which is more common). Negativity for leukocyte common antigen (LCA) and other lymphoid markers (CD3, CD20, CD7, CD4, CD79a, CD30, CD68, PAX5, ALK1, and TdT) rules out malignant lymphoma. Positivity for vimentin, CD138, MUM-1, and CD56 strongly supports a plasma cell origin, as does monoclonal kappa light-chain positivity [16].

Plasma cell tumors are well known to be radiation-sensitive, and radiotherapy represents the treatment of choice for EMP. Complete surgical excision is appropriate only when a lesion is small and localized; any role for chemotherapy remains unclear [17]. Surgery is optimal when the disease is localized and amenable to complete resection. In our case, the lesion was easily accessible, and complete surgical removal was achieved.

## Conclusion

EMP is rare, and no controlled or cohort study has examined the roles played by clinical manifestations in EMP diagnosis in adults. In adults presenting with asymptomatic tonsillar enlargement, the possibility of submucosal masses should be considered. The radiologist is encouraged to study the crypts within the palatine tonsil on postcontrast MRI, besides enlargement and signal change on T2W images. The pathologist must also carefully examine and preserve tissue for immunological characterization.
